# Project for the National Program of Early Diagnosis of Endometrial Cancer Part II

**Published:** 2015

**Authors:** RE Bohîlțea, V Ancăr, V Rădoi, F Furtunescu, LC Bohîlțea

**Affiliations:** * “Carol Davila” University of Medicine and Pharmacy, Bucharest, Romania

**Keywords:** endometrial cancer, cervical cytology, endometrial polyps, Lynch syndrome, Cowden syndrome

## Abstract

**Rationale:** Endometrial cancer recorded a peak incidence in ages 60-64 years in Romania. Since 2013, an increased trend of endometrial cancer occurrence has been registered in urban areas as compared with rural ones. Unfortunately, most of the cancer cases are diagnosed too late, in an advanced stage of the disease, resulting into diminished lifetime expectancy. The first part of the article concentrated on issues such as: the description of the study, results, and discussions regarding the study, definitions and terms, risk factors specific for endometrial carcinomas, presentation of the activities of the Program, etc.

**Objective:** Drafting a national program that will serve as an early diagnosis method of endometrial cancer. This second part of the study continues with the presentation of the activities of the Program, analyzes the human resources and materials needed to implement the Program, presents the strategies and the indicators specific for the implementation of the project.

**Methods and Results:** A standardization of the diagnostic steps was proposed and the focus was on 4 key elements for the early diagnosis of endometrial cancer: The first steps were approached in the first part of the study and the second part of the study investigated the proper monitoring of precursor endometrial lesions or cancer associated endometrial lesions and screening high risk populations (Lynch syndrome, Cowden syndrome).

**Discussion:** Improving medical practice based on diagnostic algorithms and programs improves and increases the lifetime expectancy, due to the fact that endometrial cancer is early diagnosed and treated before it causes serious health problems or even death.

**Abbreviations:** ASCCP = American Society for Colposcopy and Cervical Pathology, CT = Computerized Tomography, HNPCC = Hereditary Nonpolyposis Colorectal Cancer (Lynch syndrome), IHC = Immunohistochemistry, MSI = Microsatellites instability, MSI-H/ MSI-L = high (positive test)/ low (negative test) microsatellites instability, PCR = Polymerase chain reaction, MRI = Magnetic Resonance Imaging, SGO = Society of Gynecologic Oncologists, SHG = Sonohysterography, TVUS = Transvaginal ultrasound

The First Part of the article can be found Journal of Medicine and Life, vol. VIII, issue 3, 2015.

## The activities of the Program

**5. Investigation of the particularities/ anomalies of the cervical cytology examination**

The incidence of atypical glandular cells in the cervical cytology examination is of 0,1-2,1% and is associated in 30% of the cases with premalignant/ malignant lesions [**1**]. The sensitivity of cervical cytologic examination in detecting the endometrial carcinoma varies between 26 and 60% for the endometrioid type, up to 100% for the serous papillary carcinoma with clear cells, the sensitivity increasing with the tumor degree [**2**,**3**]. The presence of malignant cells in the cervical cytology examination represents a prediction factor for a severe myometrial invasion, ganglions metastasis, unfavorable prognosis, and a high risk of recurrence [**4**]. Rarely, the atypical glandular cells have been associated with tubal, ovarian, vaginal, colonic and breast cancer. 

The cervical cytology suspect of endometrial cancer contains the following:

- adenocarcinoma; malign cells can come from the endometrium or cervix;

- atypical endometrial cells (endometrial AGC) and atypical glandular cells (AGC) – all the subcategories besides the endometrial ones in women aged over 35 years/ with abnormal uterine bleeding/ with risk factors for endometrial cancer;

- benign/ normal endometrial cells in women aged 40 years or older, with abnormal uterine bleeding or risk factors for endometrial cancer; 

- benign/ normal endometrial cells in postmenopausal women. 

**Standard:** The initial evaluation stage contains a medical history, physical examination, and initial laboratory tests. 

**Fig. 1:**
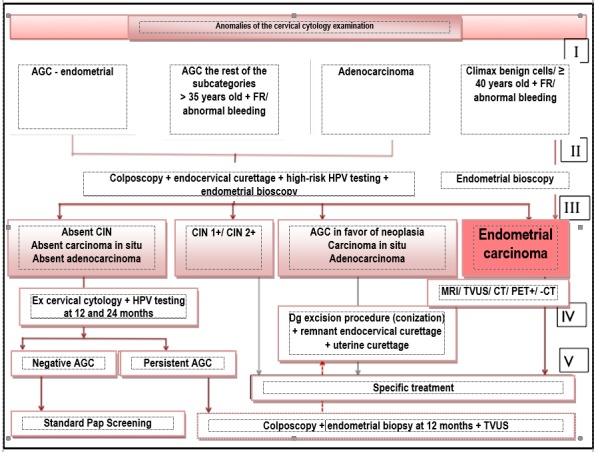
Diagnosis algorithm of the particularities/ anomalies of cervical cytology

The etiological evaluation stage for adenocarcinoma obligatorily contains atypical endometrial cells and atypical glandular cells, colposcopy with endocervical curettage, high risk HPV testing and endometrial biopsy (ASCCP). The benign/ normal endometrial cells with a supplementary exploration indication are examined by an endometrial biopsy. Patients, who have been histologically confirmed to suffer from endometrial cancer, go to stage IV and V of the diagnosis of the disease. Patients without intraepithelial cervical lesions, carcinoma in situ, and adenocarcinoma should do a co-testing (Pap + high risk HPV testing) at 12 and 24 months, whose negative result will reintegrate the patient in the standard screening program. The abnormal co-testing imposes colposcopy followed by an endometrial biopsy, which will be repeated in case a colposcopy diagnosis will not be obtained. Patients with AGC-NOS/ persistent endocervical AGC without diagnosis after repeating the colposcopy and the endometrial biopsy need conization. In the presence of symptoms, persistent endometrial AGC imposes an annual biopsy repeating and examination in order to detect a primary/ secondary tubal, ovarian, pelvic, or abdominal cancer (TVUS, colonoscopy, abdominal CT, MRI). In favor of neoplasia – AGC, the carcinoma in situ or the adenocarcinoma with an unclear diagnosis after anterior investigations need a diagnosis excision procedure (preferably conization), endocervical curettage of the remaining cervix and endometrial curettage. The lack of specification of the diagnosis imposes the ultrasound investigation of the tubes and ovaries in search of lesions at this level [**5**,**6**]. (**Fig. 1**)

Patients with positive vaginal cytology post hysterectomy for cervical or endometrial cancer must be specifically investigated in order to determine recurrence. 

**Recommendation:** Normal endometrial cells which appear in an asymptomatic premenopause woman (incidence 0,9%) are rarely associated with pathology and do not impose a further evaluation. Their appearance in women with abnormal uterine bleeding or risk factors for endometrial cancer indicates further investigations. 

**6. Diagnosis, monitorization and adequate treatment of endometrial lesions precursor/ associated to endometrial cancer**

Endometrial hyperplasia, which represents the proliferation of endometrial glands under an estrogenic stimulus, inadequately unantagonized as far as progesterone is concerned, is classified according to OMH 1994, from the point of view of glandular architecture and cytological atypia, as simple/ complex, without / with atypia, the rates of progress to endometrial cancer being of 1%, 3%, 8%, and respectively 29%, and the risk of coexistence with it being of 37%.

Endometrial polyps represent benign hyperplastic proliferations with the possibility of becoming malignant. During both premenopause and post menopause, polyps determine abnormal uterine bleedings or can have an asymptomatic evolution. Risk factors: high level of estrogen activity of exogenic or endogenic provenance, Lynch and Cowden syndromes, tamoxifen therapy. Risk factors for malignization: post menopause, presence of symptomatology, diameter of over 1,5 cm, tamoxifen therapy. Post menopause, the risk of malignization of the endometrial polyps is of 10/00 and the risk of polypoid growth is of 40/00.

The population with a high risk who should be examined in order to determine the diagnosis is represented by common categories of endometrial cancer.

**Standard:** The initial evaluation stage is made up of medical history, physical examination and initial laboratory tests. The etiological evaluation stage is based on TVUS with Doppler and instillation of saline solution, which directs the patients with a global lesion to suction biopsy or dilation and uterine curettage in cases mentioned in the diagnosis stage, and, the ones with focal lesions to hysteroscopy with the resection of the polyp and uterine curettage post hysteroscopically. TVUS, sonohysterography and hysteroscopy are methods with a similar efficiency in diagnosing and monitoring the endometrial polyps (91%, 95% and respectively 90% sensitivity, 90%, 92% and respectively 93% specificity). The histopathological examination establishes the diagnosis of endometrial polyp, endometrial hyperplasia, and its type according to the classification of the Order of the Ministry of Health (OMH). Women with atypical hyperplasia as a result of suction biopsy must be examined by dilation and biopsy curettage in order to exclude a coexistent endometrial cancer. In case uterine curettage has a less severe lesion/ absence of a lesion as a result, the management will be according to the most severe lesion (atypical hyperplasia). Extrafascial total hysterectomy is the chosen treatment for patients with endometrial hyperplasia with atypia, who do not plan a new pregnancy. The element is intraoperatory evaluated to exclude the endometrial carcinoma. Post menopause hyperplasias with atypia benefit from total hysterectomy with a bilateral adnexectomy. Premenopausal hyperplasias with atypia treated by hysterectomy have a controversial indication of bilateral adnexectomy, the advantage of adnexectomy being the avoidance of reintervention in case endometrial cancer also exists, the disadvantage being the inutility of the procedure for 50-80% of the patients with atypical hyperplasia without endometrial cancer. The patients with a benign result or endometrial hyperplasia without atypia on frozen sections do not need bilateral adnexectomy in the absence of other indications. The hormonal substitution therapy in young patients with a total post hysterectomy and bilateral adnexectomy does not raise the risk of recurrence in women with endometrial cancer. Progestative therapy represents an option for the women with hyperplasia with atypia who want to preserve fertility or who cannot undergo surgery. Their monitoring by endometrial biopsy at every 3 months is compulsory. Pregnancy can be obtained after the regression of atypical hyperplasia; the maintenance therapy, whose purpose is the prevention of recurrences, is maintained until a pregnancy occurs, together with an endometrial biopsy, which is repeated at 6-12 months. The maintenance of regression demonstrated by 2 normal biopsies place the patient in the biopsy-monitoring program at 1-2 years. The failure of medication therapy (persistent lesion/ recurrence/ progression to endometrial cancer) represents a clear indication for hysterectomy. Stopping the family planning recommends hysterectomy. Postmenopausal women under progestative therapy for hyperplasia with atypia are recommended an endometrial biopsy repeated at 6-12 months on an indefinite period of time or until hysterectomy can be performed. 

Endometrial hyperplasia without atypia benefits from progestative therapy on the expense of surgery. Control endometrial biopsy is indicated at 3-6 months. The persistence of the lesion imposes the change of doses/ diet/ food. Regression is followed by control maintenance therapy by an endometrial biopsy at 6-12 months. The expectant management is recommended to patients with hyperplasia without asymptomatic atypia during premenopause or post menopause, patients with counter indications for the administration of progestatives and patients who refuse or cannot tolerate therapy. The monitorization by endometrial biopsy is indicated at 3-6 months until the lesion’s regression. The progression to hyperplasia with atypia/ endometrial cancer imposes hysterectomy with the stipulations of the diagnosis protocol previously established.

Progestative therapy presupposes the oral administration of acetate medroxyprogesterone/ megestrol in doses, continuously at 3-9 months or cyclically at 12-14 days/ month, depot medroxyprogesterone acetate intramuscularly administered at 3 months, microdose natural progesterone intravaginally administered continuously or cyclically at 12-14 days/ month, intrauterine device with a slow discharge of LNg20 progesterone, alone or associated with oral progestative therapy.

**Fig. 2:**
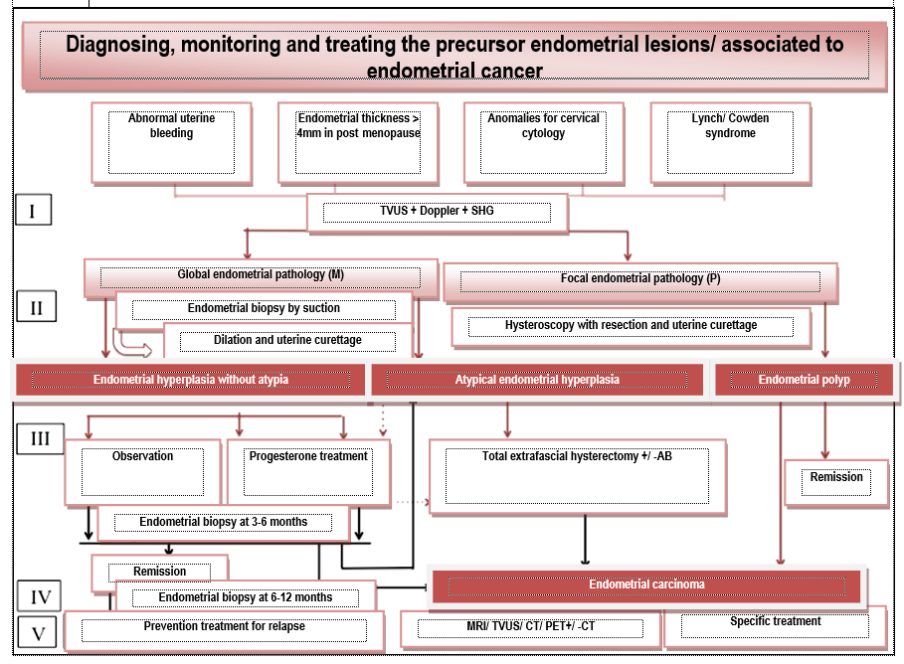
Diagnosis algorithm, monitorization and treatment of the lesions precursor to endometrial cancer

The diagnosis of endometrial polyps is histopathological or of exclusion of malignity. The hysteroscopic polypectomy represents the treatment of choice for endometrial polyps, followed by biopsy uterine curettage of the entire cavity. When diagnosed hysteroscopy is followed by the excision of the polyps through uterine curettage, after the procedure, a control hysteroscopy must be performed. If hysteroscopy is unavailable, polypectomy can be realized by dilation and uterine curettage, with the risk of underdiagnosing the small dimensions focal lesions and of other structural anomalies. The presence of the polyp on fragments resulted from the suction biopsy needs polypectomy in order to eliminate the symptoms and the exclusion of malignity. The symptomatic polyps must be excised especially in premenopause women with risk factors for hyperplasia or endometrial cancer. In the absence of symptomatology, the bigger, multiple, prolab polyps of 1.5 cm or the ones of infertile patients must be excised. In post menopause women, polyps should obligatorily be excised. The recurrence of polyps is reduced; no systematic follow up and tamoxifen treatment is recommended after polypectomy (**Fig. 2**).

**Recommendation:** The development of an endometrial hyperplasia in post menopause without obvious sources of hyperestrogenism requires investigations in order to trace an estrogen-secreting tumor. The weight loss is recommended in obese women. 

**Option:** Oestroprogestative contraceptives are indicated in simple hyperplasia without atypia when contraception is concomitantly required or in cases of chronic anovulation. The ovulation inductors are indicated in patients with hyperplasia without atypia who wish to get pregnant. Hysteroscopic endometrial ablation is indicated in hyperplasia without atypia. The alternative pharmacological options that are being currently evaluated are Danazol, GnRH+/- LNg 20 agonists/ antagonists and Anastrozole – aromatase inhibitors. 

**7. Screening of the high-risk population (Lynch syndrome, Cowden syndrome)**

**Lynch syndrome** represents 2-5% of the total endometrial carcinomas and 3% of the total colonic adenocarcinomas. The risk of developing endometrial cancer during lifetime of women with Lynch syndrome is of 27-71% compared with 2,6% in the general population and the medium age of diagnosis decreases from 60-64 years to 46-54 years. In the Lynch syndrome case, cancer can be localized as primary unique/ synchronous/ metachronous tumors: colorectal, endometrial, ovarian, upper urinary tract, gastric, small intestine, biliary/ pancreatic, cerebral (gliomas), of sebaceous glands and keratoacanthoma. Lynch syndrome is an autosomal dominant medical condition, whose susceptibility is genetically transmitted by the development of cancer and determined by a germinal line mutation, which appeared at the level of one of the genes responsible for DNA coupling error repair (MMR genes), or by the loss of MSH2 expression due to a deletion of the EPCAM gene. MMR genes associated to Lynch syndrome are the following: MLH1 which is localized on 3p21 chromosome (32% of the cases), MSH2 which is localized on 2p16 chromosome (39% of the cases), MSH6 which is localized on 2p16 chromosome (15% of the cases, with a high risk of endometrial cancer) and PMS2 which is localized on 7p22 chromosome (14% of the cases). 

The identification of the population with a high risk of developing Lynch syndrome: 

Amsterdam II Criteria [**7**]:

- Minimum 3 relatives cu Lynch-associated cancer (colorectal, endometrial, of small intestine, urethral or of renal pelvis) 

- One of the relatives must be a Ist degree relative with the other two

- Minimum 2 successive generations affected 

- Minimum a case of cancer diagnosed under the age of 50 years

- Familial adenomatous polyposis must be excluded from the category of colorectal cancer 

- Tomors should be examined from a histopathological point of view

The revised Bethesda Criteria [**8**] for MSI testing of colorectal tumors are the following: 

- Colorectal cancer diagnosed in a patient under 50 years old 

- Presence of synchronous/ metachronous colorectal or Lynch-associated tumors, regardless the age 

- Colorectal cancer with a histology of MSI-H type diagnosed in a patient under 60 years old (lymphocyte tumor infiltrate, Crohn-like lymphocyte reaction, mucinous differentiation/ signet ring carcinoma, medullary growth pattern) 

- Colorectal cancer diagnosed in a patient with one or more Ist degree relatives with Lynch-associated tumors, one of the cancers being diagnosed under the age of 50 years

- Colorectal cancer diagnosed in a patient with two or more Ist/ IInd degree relatives with Lynch-associated tumors, regardless the age

SGO Guidelines [**9**] regarding the evaluation of the Lynch syndrome: 

- Risk of Lynch syndrome > 20-25% (recommended genetic testing)

• Endometrial/ ovarian cancer with a synchronous/ metachronous colorectal cancer, the first one being diagnosed under the age of 50 years

• Endometrial or colorectal cancer which meets the Amsterdam II Criteria

• Ist/ IInd degree relative with a diagnosis of one MMR gene mutation 

- Risk of Lynch syndrome > 5-10% (useful genetic testing) 

• Endometrial/ ovarian cancer with synchronous/ metachronous colorectal cancer or another type of Lynch-associated cancer – regardless the age of appearance of the first cancer 

• Endometrial or colorectal cancer diagnosed under the age of 50 years 

• Endometrial or colorectal cancer with two or more Ist/ IInd degree relatives with Lynch-associated cancers 

• Ist/ IInd degree relatives who meet the afore-mentioned criteria of the risk-group.

The certainty diagnosis of Lynch syndrome is based on the genetic determination of the germinal line mutation of one of the MMR or EPCAM genes. Due to the fact that the testing of the germinal line of all the patients suspect of Lynch syndrome is very expensive, a sequential genetic evaluation, which starts with PCR tumor testing for MSI (MSI-H = positive test) and/ or by immunohistochemistry for the MMR genes expression (loss of the proteins color = positive test), should be undergone [**10**]. Most of the experts recommend the MSI/ IHC testing of all the colorectal or endometrial cancers. 

**Standard:** MSI/ IHC testing is mandatory for some categories: 

- endometrial cancer diagnosed before 50 years old;

- endometrial cancer with lymphocyte tumor or peritumoral infiltrate or tumors which are not differentiated from a histologic point of view or their origin is in the uterine inferior segment, diagnosed in a woman under 60 years old; 

- Ist degree relatives of the patients diagnosed with MMR/ EPCAM genes mutations; 

- medical family history of cancer, meeting the Amsterdam II Criteria or the revised Bethesda Guidelines. 

Negative MSI and IHC invalidate the affiliation of the tumor to Lynch syndrome. Positive MSI patients or with the altering of MMR genes on IHC, patients whose MSI/ IHC tumor testing is unavailable, but in whom there is a strong clinical suspicion (Bethesda Criteria), as well as patients who meet the Amsterdam II criteria in the absence of tumor testing, need the testing of germinal lines in order to determine MMR/ EPCAM mutations.

**Fig. 3:**
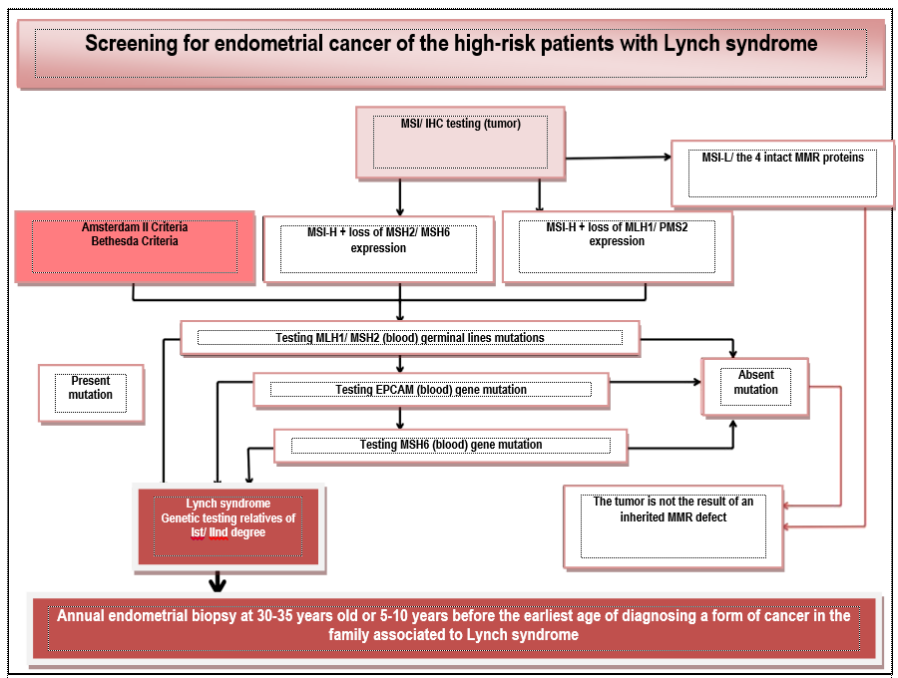
Diagnosis algorithm and observation of Lynch syndrome by screening

The management of the patients with Lynch syndrome includes observation by screening, chemoprevention, and surgery in order to reduce risk. Screening for endometrial cancer consists of an endometrial biopsy annually performed starting with 30-35 years old or 5-10 years before the earliest age for the diagnosis of a cancer form associated to Lynch (HNPCC) syndrome in the family [**12**,**13**]. After the family planning, patients with a certain genetic diagnosis of Lynch syndrome, are recommended total hysterectomy with bilateral adnexectomy in order to reduce the risk (Cancer Genetics Consortium) [**12**] (**Fig. 3**).

**Cowden syndrome** is a genetic affection, which has an autosomal dominant transmission, its base being the mutation of PTEN suppressor tumor gene, localized on chromosome 10q23. The risk of developing endometrial cancer in these patients during lifetime is of 13-28%, cases of endometrial cancer being also reported in adolescents. 

The clinical diagnosis criteria of Cowden syndrome [**11**] (adopted by the National Comprehensive Cancer Network) are the following:

Major criteria:

- Breast cancer 

- Endometrial cancer 

- Thyroid (follicular) cancer 

- Gastrointestinal hamartomas (including ganglioneuromas, excluding hyperplastic polyps; ≥ 3)

- Lhermitte-Duclos disease (in adults)

- Macrocephaly (≥ 97 percent: 58 cm for women, 60 cm for men)

- Macular pigmentation of the pineal gland 

- Multiple cutaneous-mucous lesions (any of the following): 

• Multiple trichilemmomas (≥ 3, at least one was proved in biopsy) 

• Acral keratosis (≥ 3 palmar-plantar keratosis lesions and/ or acral keratosis papules)

• Cutaneous-mucous neuromas (≥ 3)

• Buccal papillomas (especially lingual and gingival), multiple (≥ 3)

• Biopsy/ dermatological diagnosis proof 

Minor criteria:

- Autistic type specter of affections 

- Colon cancer 

- Esophageal glycogenic acanthosis (≥ 3)

- Lipomas (≥ 3)

- Mental retardation (IQ < 75)

- Carcinoma of the renal cells 

- Testicular lipomatosis 

- Thyroid cancer (papillary/ follicular type of the papillary one) 

- Structural thyroid lesions (adenomas, multinodular goiter) 

- Vascular anomalies (including multiple development intracranial venous anomalies). 

The individual operational diagnosis (any of the following): 

1. three or more major criteria, among which one is obligatorily macrocephaly, Lhermitte-Duclos disease or gastrointestinal hamartomas 

2. two major criteria and three minor criteria 

The operational diagnosis in families with a person who meets the individual diagnosis criteria or with a PTEN genetic mutation diagnosis: 

1. any two major criteria with/ without minor criteria 

2. one major criterion and two minor criteria

3. three minor criteria. 

**Standard:** The testing criteria for Cowden syndrome (National Comprehensive Cancer Network) are the following:

- A person who comes from a family with a known PTEN mutation 

- Individual diagnosis criteria for Cowden syndrome: Bannayan-Riley-Ruvalcaba syndrome/ Lhermitte-Duclos adult disease/ autism and macrocephaly/ two or more trichilemmomas proven by biopsy/ two or more major criteria among which one is macrocephaly/ three major criteria without macrocephaly/ one major criteria and three or more minor criteria/ four or more minor criteria. 

- Persons with high risk: persons with relatives diagnosed with Cowden syndrome/ PTEN hamartoma tumor syndrome/ Bannayan-Riley-Ruvalcaba syndrome untested genetically and which presents any major criteria or two minor criteria. 

The persons who meet the testing criteria are directed to undergo a genetic consultation and testing (sequencing the entire coding area, analyses for deletion/ duplication) for PTEN mutation. 

Patients with Cowden syndrome must be informed regarding the risks and are advised to promptly present to a specialty examination in case abnormal uterine bleedings appear. The hysterectomy option must be discussed. They must be completely physically examined annually. Screening by endometrial biopsy is recommended starting with 35-40 years old or 5 years earlier as compared to the age the first case of endometrial cancer was diagnosed in a family member. Postmenopausal women are recommended annual transvaginal ultrasound. 

**8. The awareness of the women regarding the bleeding anomalies and addressing to the family physician**

On the occasion of the annual/ triennial visit to the physician for the evaluation of the health state, the family physician must perform an anamnesis of the patient according to her age, the variables with concern to the menses/ menopause and the possibility of some bleedings. Moreover, the family physician must counsel the patient regarding the recognizing of a pathological bleeding and the importance of immediately addressing the doctor. On the visit, the family physician can give the patient a flyer with general information regarding abnormal bleeding. 

**Human resources and materials necessary to implement the program **

**Strategies for the implementation of the program**

• checklist/ prioritized schemes of specific interventions; 

• identifying the problems in adopting the recommendations and offering personalized solutions; 

• integrating the recommendations in the current medical practice conditions in gynecology; 

• identifying the systems and technical solutions of compliance to the application of recommendations of the program;

• developing and creating interdisciplinary teams (clinical epidemiology, implementation science, computerized technology) for the study of the optimum application methods. 

The studies regarding the implementation of guidelines and recommendations have shown a raise in their acceptance and application, in the conditions of their substantiation by clear profs and the compatibility with the current rules and practices, in the absence of the need of acquiring new technical abilities, in the absence of controversies generating and in the conditions of a better clinical application.

The tools proposed were generated based on the personal clinical experience and the literature review. In order to turn into medical practice algorithms a group of experts should validate them. 

Once validated, the tools can be shared with OG specialists and the family physicians (the specific information will be kept for each medical assistance level). 

The successful criteria for the implementation of the adequate medical practice are the following: 

- systematic sharing with the physicians (all the OG specialists and the family physicians should receive the information) 

- organizing discussions/ professional forums for the implementation of the new practices 

- evaluating the implementation of the practices 

**Monitoring indicators specific for the implementation of the project**

**Administrative issues**

Patients with abnormal uterine bleeding and the ones with particularities/ anomalies of the cervical cytology examination can address the family physician or the gynecologist. The initial evaluation stage can be performed in any medical unit by the family physician or the gynecologist. 

The cases of patients with abnormal uterine bleeding or the ones with particularities/ anomalies of the cervical cytology examination, who are suspected of endometrial cancer, based on the initial evaluation stage, cases with precursor endometrial lesions/ associated with endometrial cancer which were diagnosed and treated, being in the monitoring period and the high-risk population (Lynch syndrome, Cowden syndrome), are evaluated by the gynecologist in outpatient or in a hospital, which must have a department of imagistic explorations and a laboratory of pathological anatomy. The laboratory of pathological anatomy must ensure the possibility of undergoing paraffin, ice, and immunohistochemistry examinations. 

The cases of patients with acute abnormal uterine bleeding or hemodynamically unstable are evaluated by the gynecologist in an emergency hospital unit. 

The patients diagnosed with endometrial cancer must be guided to do pre-therapeutic psychological counseling. 

Patients with endometrial cancer, which appeared while suffering from Lynch syndrome (HNPCC) or Cowden syndrome, must be guided, together with the family members, towards undergoing a genetic consultation and genetic examination specific for the mutations of MMR and PTEN genes.

The conduct of the patients diagnosed with endometrial cancer is established by an interdisciplinary collaboration between surgeon, oncologist, and pathologist. 

Patients diagnosed with endometrial cancer will be surgically treated in a hospital unit by a gynecologist who has a gynecology complementary studies certificate or by a mixed team made up of a gynecologist-oncological surgery specialist.

The monitorization indicators specific to the program are the following: 

a. number of OG who have received the information and the tools (quarterly, annually) 

b. number of family physicians who have received the information and the tools (quarterly, annually) 

c. number of patients with a correct medical history regarding the abnormal bleedings at the MF level (quarterly, annually) 

d. % of patients counseled at the MF level regarding the abnormal bleedings from the total eligible patients recorded (annually) 

e. number of patients who are at risk and who take part in screening for endometrial cancer (annually) 

The evaluation indicators should reflect the degree in which the proposed tools determine modifications in the management of the cases and should be systematically evidenced (for example annually) at the level of each provider of medical services.

Some proposed indicators are the following: 

a. % of cases with abnormal bleeding, investigated according to the practice algorithm (annually)

b. % of cases with anomalies of the cervical cytology examination, investigated according to the practice algorithm (annually)

c. % of cases with precursor endometrial lesions/ associated to endometrial cancer, investigated according to the practice algorithm (annually) 

d. % of cases with Lynch and Cowden syndrome, identified before the appearance of endometrial cancer (annually) 

e. % of cases of endometrial cancer discovered in stage I of the disease 

f. post-diagnosis survival of patients with endometrial cancer. 
